# The Effect of Aromatic Plant Extracts Encapsulated in Alginate on the Bioactivity, Textural Characteristics and Shelf Life of Yogurt

**DOI:** 10.3390/antiox12040893

**Published:** 2023-04-06

**Authors:** Liliana Popescu, Daniela Cojocari, Aliona Ghendov-Mosanu, Ildiko Lung, Maria-Loredana Soran, Ocsana Opriş, Irina Kacso, Alexandra Ciorîţă, Greta Balan, Adela Pintea, Rodica Sturza

**Affiliations:** 1 Faculty of Food Technology, Technical University of Moldova, 9/9 Studentilor St., MD-2045 Chisinau, Moldova; 2 Department of Preventive Medicine, “Nicolae Testemitanu” State University of Medicine and Pharmacy, 165 Stefan cel Mare Bd., MD-2004 Chisinau, Moldova; 3 Department of Physics of Nanostructured Systems, National Institute for Research and Development of Isotopic and Molecular Technologies, 400293 Cluj-Napoca, Romania; 4 Faculty of Biology and Geology, Babes-Bolyai University, 5–7 Clinicilor, 400006 Cluj-Napoca, Romania; 5 Faculty of Veterinary Medicine, University of Agricultural Sciences and Veterinary Medicine, 3–5 Calea Manastus St., 400374 Cluj-Napoca, Romania

**Keywords:** summer savory, rosemary, extraction, encapsulation, functional foods, concentrated yogurt

## Abstract

The article investigated the antioxidant and antimicrobial activity of extracts from two aromatic plants—*Satureja hortensis* L. (SE) and *Rosmarinus officinalis* L. (RE), encapsulated in alginate, on—yogurt properties. The encapsulation efficiency was controlled by FTIR and SEM analysis. In both extracts, the individual polyphenol content was determined by HPLC–DAD–ESI-MS. The total polyphenol content and the antioxidant activity were spectrophotometrically quantified. The antimicrobial properties of SE and RE against gram-positive bacteria (*Bacillus cereus*, *Enterococcus faecalis*, *Staphylococcus aureus*, *Geobacillus stearothermophilus*), gram-negative bacteria (*Escherichia coli*, *Acinetobacter baumannii*, *Salmonella abony*) and yeasts (*Candida albicans*) were analyzed in vitro. The encapsulated extracts were used to prepare the functional concentrated yogurt. It was established that the addition of 0.30–0.45% microencapsulated plant extracts caused the inhibition of the post-fermentation process, the improvement of the textural parameters of the yogurt during storage, thus the shelf life of the yogurt increased by seven days, compared to the yogurt simple. Mutual information analysis was applied to establish the correlation between the concentration of the encapsulated extracts on the sensory, physical-chemical, and textural characteristics of the yogurt.

## 1. Introduction

Yogurt is considered one of the most popular fermented dairy products [1]. Consumers demand yogurt not only because of the bioavailability of essential nutrients resulting from yogurt’s bacterial activity [2] but also for the wide product variations that are available in terms of texture and flavor. Concentrated yogurt is a fermented milk in which the protein content has been raised to a minimum of 5.6% [3]. This type of yogurt has gained increased consumer interest due to the improved taste and texture as well as the health benefits of milk proteins [4,5]. In addition, concentrated yogurt could be beneficial in calorie-restricted diets because energy intake from protein has a greater effect on satiety than fat or carbohydrate intake [6]. Consequently, concentrated yogurt could be enriched with various bioactive ingredients such as probiotics, phenolic compounds, carotenoids, polyunsaturated fatty acids, dietary fiber, vitamins, mineral salts, and others. [7,8]. Phenolic compounds have demonstrated antioxidant, antimicrobial, and anti-inflammatory activity and exhibit anticancer effects [9], including phenolic compounds from aromatic plants. Therefore, phenolic compounds have been widely proposed as fortification agents for the production of functional foods, especially during the production of fermented dairy products [8]. Despite this fact, their application and use in food are reduced due to susceptibility to bitter or astringent taste, and low bioavailability [10]. The nano/microencapsulation approach facilitates the delivery of phenolic substances in different food matrices, leading to improved stability and solubility of bioactive compounds during processing, storage, or gastric digestion of the product [7]. Microencapsulation consists of the protection of different food components or functional constituents against outside factors such as temperature, oxygen, light, humidity, or interactions with food constituents, for example, proteins [11,12]. The microencapsulation mechanism involves covering bioactive substances with a polymeric or non-polymeric material, promoting their controlled release under special conditions [13]. Different coating materials are used depending on their rheological properties, and their ability to disperse the active compound and stabilize it. Sodium alginate is a frequently used coating material, the encapsulation of bioactive compounds inside being achieved through the cross-linking process in the presence of bivalent ions. The structure formed can withstand acidic environments and leads to the controlled release of bioactive compounds [14]. 

The objective of this study was to investigate the antioxidant and antimicrobial activity of extracts from two aromatic plants (rosemary and summer savory) and to examine the functional properties of plant extracts encapsulated in alginate. The study was designed to investigate the possibility of developing functional concentrated yogurt fortified with encapsulated phenolic compounds with bioactivity and improved sensory and textural characteristics.

## 2. Materials and Methods

### 2.1. Materials

The plant material used in this study consisted of leaves collected from summer savory (*Satureja hortensis* L.) and rosemary (*Rosmarinus officinalis* L.) plants harvested during August 2022 from AromeNature, Peticeni commune, Calarasi, Republic of Moldova (47°14′10″ N 28°12′31″ E). Plant leaves were dried at 60 ± 1 °C until the moisture content dropped to 5.2% and stored in dark packages at room temperature until extraction. Standardized milk (2.5% fat, 2.9% protein, and 4.6% lactose) and cream (10.0% fat, 3.0% protein, and 4.2% lactose) according to the information on the label, were purchased from JLC, Republic of Moldova. Milk protein concentrate (85% protein, 1.6% fat, and 4.3% lactose) and whey protein concentrate (82.8% protein, 6.2% fat, and 4.7% lactose) according to the information on the label (Lactoprot, Kaltenkirchen, Germany). Freeze-dried, direct vat set (FD–DVS) yogurt starter culture contains *Streptococcus thermophilus*, *Lactobacillus delsbrueckii* subsp. *bulgaricus*, *Lactobacillus acidophilus*, and *Bifidobacterium* (YAB 352B, Sacco, Italy).

### 2.2. Reagents

Folin–Ciocalteu reagent, sodium carbonate (Na_2_CO_3_), DPPH (2,2′-diphenyl–picrylhydrazyl), calcium chloride, and sodium alginate were purchased from Merck (Darmstadt, Germany), gallic acid equivalent (GAE) was acquired from Sigma-Aldrich (Darmstadt, Germany), absolute ethanol and methanol were supplied from Chimopar (Bucharest, Romania). Acetonitrile, HPLC–gradient, and ultrapure water were produced with a Direct-QR 3 UV Water Purification System, Merck (Darmstadt, Germany). The pure standard of chlorogenic acid (>98% HPLC), luteolin (>99% HPLC), gallic acid (>99% HPLC), and cyanidin (>99% HPLC), were purchased from Sigma (Sigma-Aldrich, St. Louis, MO, USA). All reagents used in this study were of analytical grade.

### 2.3. Extraction of Polyphenolic Compounds

The polyphenolic extracts were obtained by sonication (Elma Transonic T 310 at 35 kHz and installed power of 95 W) for 30 min at room temperature of a mixture containing milled (Hausberg electric grinder, power 250 W) dried summer savory or rosemary leaves and 60% (*v*/*v*) ethanol in a ratio of 1:10 (*w*/*v*). In the end, the mixture was centrifugated for 10 min at 7000 rpm in order to separate the supernatant. The obtained extract was kept in the refrigerator at 4 ± 1 °C until its analysis.

### 2.4. Characterization of Obtained Extracts

#### 2.4.1. Total Polyphenol Content

The analysis of total polyphenols was carried out according to the Folin–Ciocalteu method [15]. In consequence, 5 mL of double distilled water, 1 mL of extract, and 0.5 mL of Folin–Ciocalteu reagent were mixed in a 10 mL graduated flask. After the mixture was allowed to stand for 3 min, 1.5 mL of Na_2_CO_3_ (5 g/L) was added and completed until 10 mL with double distilled water. The flask with the resulting mixture was kept for 16 min at 50 °C (in a water bath), after which it was allowed to cool to room temperature. After cooling, the absorbance of the mixture was read relative to the control sample (double distilled water) at a wavelength of 765 nm using a UV–VIS T80 spectrophotometer (PG Instruments Limited, Leicestershire, UK). The total polyphenol concentration of the samples was calculated using a standard gallic acid equivalent (GAE) curve. The curve was drawn for the range of 0.002–0.8 mg/mL, the solutions being obtained by successive dilutions from a standard solution of 1 mg/mL.

#### 2.4.2. HPLC–DAD–ESI-MS Analysis of Polyphenols

Analysis was carried out using an Agilent HP-1200 liquid chromatograph equipped with a quaternary pump, autosampler, DAD detector, and MS-6110 single quadrupole API–electrospray detector (Agilent Technologies, Santa Clara, CA, USA). The positive ionization mode was applied to detect the phenolic compounds; different fragment, in the range of 50–100 V, was applied. The column was an Eclipse XDB-C18 (5 μm; 4.5 × 150 mm i.d.) from Agilent. The mobile phase was (A) water acidified by acetic acid 0.1% and (B) acetonitrile acidified by acetic acid 0.1%. The following multistep linear gradient was applied: start with 5% B for 2 min; from 5% to 90% of B in 20 min, hold for 4 min at 90% B, then 6 min to arrive at 5% B. The total time of analysis was 30 min, the flow rate was 0.5 mL/min, and the oven temperature was 25 ± 0.5 °C. Mass spectrometric detection of positively charged ions was performed using the Scan mode. The applied experimental conditions were gas temperature 350 °C, nitrogen flow 7 L/min, nebulizer pressure 35 psi, capillary voltage 3000 V, fragmentor 100 V, and *m*/*z* 120–1200. Chromatograms were recorded at wavelength λ = 280, 340, and 520 nm and data acquisition were done with the Agilent ChemStation software. The content of specific polyphenols was determined by comparison of retention times and peaks with the ones from the chromatogram of a synthetic mix containing the standards listed in Table 1.

#### 2.4.3. Determination of the Antioxidant Activity of Extracts

The antioxidant activity of the plant extracts was determined according to the DPPH method of Brand-Williams and collaborators [16], slightly modified. Therefore, 0.001 mL of the extract was added to 3.9 mL of DPPH radical solution (0.005 g/200 mL methanol). The resulting mixture was left in the dark for 10 min, after which its absorbance was read at 515 nm compared to the blank consisting of 0.001 mL of extract added to 3.9 mL of methanol, with the UV–VIS T80 spectrophotometer. (PG Instruments Limited, Leicestershire, UK). The results were calculated from the Trolox calibration curve, drawn for concentrations in the range of 0.004–3.2 mM, and the results were expressed in mM TE/g DW.

#### 2.4.4. Determination of the Antimicrobial Activity of Extracts

##### Preparation of Inoculum

For this analysis and for the preparation of microencapsulated plant extract, the alcohol was evaporated from the plant extracts in a Heidolph Rotavapor (Heidolph Instruments GmbH & Co, Schwabach, Germany) at a temperature of 40 °C and a pressure of 175 mbar.

The antimicrobial properties of extracts were tested against gram-positive bacteria (*Staphylococcus aureus* ATCC 25923, *Bacillus cereus* ATCC 11778, *Enterococcus faecalis* ATCC 19433, *Geobacillus stearothermophilus* ATCC 7953), gram-negative bacteria (*Escherichila coli* ATCC 25922, *Acinetobacter baumannii* ATCC^®^ BAA-747, *Salmonella abony* NCTC 6017), as well as one pathogenic fungus *Candida albicans* ATCC 10231. All test culture was purchased from American Type Culture Collection (ATCC), except *Salmonela abony*, which was obtained from The National Collection of Type Cultures (NCTC; UK). Bacterial cultures were pre-cultured in Mueller Hinton broth overnight at 37 °C. Each strain was adjusted to a concentration corresponding to the 0.5 McFarland standard [17]. The fungal inoculum was prepared from the 48-hour culture grown in Potato dextrose broth [18].

##### Antimicrobial Screening

To determine the antimicrobial effect of the extracts, we screened them by the well diffusion method [19,20]. Wells were made in Mueller Hinton agar plates using a sterile metal punch (6 mm in diameter). The plates were inoculated with a sterile swab moistened with microbial suspension according to the 0.5 Mac Farland turbidity standard. Then, 100 µL of plant extract was added to each well. The plates were introduced in the refrigerator for 30 min to allow the extracts to diffuse well into the agar, then incubated at 37 °C for 18 h. Antimicrobial activity was detected by measuring the zone of inhibition (including the diameter of the wells) after the incubation period. DMSO (Dimethyl Sulfoxide) at a concentration of 10% was employed as a negative control.

##### Determination of Minimal Inhibitory Concentration and Minimum Bactericidal/Fungicidal Concentration

The Minimal Inhibitory Concentration (MIC) and Minimum Bactericidal/Fungicidal Concentration (MBC/MFC) of plant extracts were determined by the dilution method in liquid media according to CLSI (Clinical and Laboratory Standards Institute of the United States of America) [21].

Serial two-fold dilutions of plant extracts in concentrations ranging from 90 mg/mL to 0.351 mg/mL with adjusted bacterial concentration (10^8^ CFU/mL, 0.5 McFarland’s standard) were used to determine MIC in Mueller Hinton broth. The control contained only inoculated broth with microorganisms and was incubated at 37 °C for 24 h.

The lowest concentrations of test samples which did not show any visible growth of test organisms after macroscopic evaluation were determined as MICs, expressed in mg/mL.

After the MIC determination of the plant extracts, aliquots of 50 μL from all the tubes which showed no visible bacterial/fungal growth were inoculated on Mueller Hinton agar plates and incubated at 37 °C for 24 h. MBC/MFC is considered the lowest concentration of plant extract that killed at least 99.9% of the initial inoculums. All assays were performed in triplicate. DMSO served as a control.

### 2.5. Preparation and Characterization of Microencapsulated Plant Extracts

Sodium alginate beads were prepared by a slightly modified method by Rijo et al. [22]. An amount of 0.6 g of sodium alginate (Alg) was stirred with 20 mL of ultrapure water for 1 h at 400 rpm at 40 °C, then cooled to room temperature, when 10 mL of extract was added under stirring. The mixture was stirred for 10 min and the resulting solution was added by means of a syringe to a solution of 0.2 M CaCl_2_. The addition was carried out for 20 min under continuous stirring, after which the mixture was left to stir for another 15 min, then it was washed three times with ultrapure water. The obtained beads were lyophilized.

For the morphological examination of the microencapsulated rosemary extract (MRE) and microencapsulated summer savory extract (MSE) a scanning electron microscope SEM Hitachi SU8230 (Hitachi, Tokyo, Japan) was used.

FT–IR spectra were recorded using a JASCO 6100 FTIR spectrometer ((JASCO International Co., Ltd., Tokyo, Japan) in the 4000 to 400 cm^−1^ spectral range, with 4 cm^−1^ resolution by the KBr pellet technique. Each sample has been dispersed in about 300 mg of anhydrous KBr mixed in an agate mortar. The pellets were obtained by pressing the mixture into an evacuated die. The spectra were collected and analyzed with Jasco Spectra Manager v.2 software.

### 2.6. Encapsulation Efficiency

The encapsulation efficiency was performed according to Pasukamonset et al. [23]. Thus, 10 mg of beads were sonicated for 30 min in 5 mL of sodium citrate (5% *w*/*v*), then the mixture was centrifuged for 10 min at 3000 rpm. The encapsulation efficiency was calculated according to the following equation:

(1) $$EE\;\left(\%\right)=\frac{TPC_{beads}}{TPC_{extract}}\times 100,\;\;$$

where *TPC_beads_* represents the total content of polyphenols encapsulated in beads and *TPC_extract_* represents the total content of polyphenols of the extracts used in the process of obtaining the beads.

### 2.7. Preparation and Characterization of Concentrated Yogurt with Microencapsulated Plant Extracts

#### 2.7.1. Preparation of Concentrated Yogurt with Microencapsulated Plant Extracts

Two concentrated yogurt sample lines were obtained: one with 0.15, 0.30, 0.45, and 0.60% MSE (0.15%CYMSE, 0.30%CYMSE, 0.45%CYMSE, and 0.60%CYMSE) and the other with 0.15, 0.30, 0.45 and 0.60% MRE (0.15%CYMRE, 0.30%CYMRE, 0.45%CYMRE and 0.60 %CYMRE). Cream (87.5%) was mixed with standardized milk (9.2%), milk protein concentrate (2.3%), and whey protein concentrate (1.0%) the resulting mix was pasteurized at a temperature of 85 ± 1 °C for 10 min, cooled to a temperature of 39 ± 1 °C, then different concentrations of microencapsulated plant extracts (0.15%, 0.30%, 0.45% and 0.60%, relative to milk) were added and inoculated with the starter culture (0.02 U/1000 mL). The milk was mixed, dispensed into glass containers (125 g), and fermented at temperatures of 39 ± 1 °C to pH 4.60 ± 0.01. After fermentation, the yogurt samples were cooled to a temperature of 4 ± 1 °C. Microencapsulated plant extracts are added to milk after pasteurization and cooling at a temperature of 39 ± 1 °C to prevent the loss of biological substances from the microcapsules. Recontamination of milk after pasteurization is excluded, and microencapsulated lyophilized plant extracts are preserved and introduced into milk in compliance with the hygiene requirements.

#### 2.7.2. Physicochemical Analysis

Fat content was determined by gravimetric methods [24]. The total solids content was determined by ISO 6731:2010 [25]. Protein content was determined by Kjeldahl methods [26]. The pH was measured with a Titrator SI Analytics TitroLine^®^ 5000 (Xylem Analytics, Letchworth, UK), at 20 °C. Physicochemical properties, except for pH, were determined only on the first day of storage.

#### 2.7.3. Sensory Analysis

The sensory analysis of the yogurt samples was determined according to Popescu et al. [27]. The sensory analysis of the concentrate yogurt samples was determined at different storage periods (1, 8, 15, 23, and 30 days).

#### 2.7.4. Texture Profile Analysis

Texture Profile Analysis (TPA) of the yogurt samples was assessed with a TA.HD Plus C Texture Analyzer (Stable Micro Systems, Godalming, UK) using an extrusion cone P/40C, according to Yilmaz-Ersan et al. [28].

#### 2.7.5. Total Polyphenolic Content and Antioxidant Activity

##### In Vitro Digestion Model

In vitro digestion of yogurt samples was performed according to the INFOGEST 2.0 protocol, described by Brodkorb et al. [29]. At the end of the digestion process, samples were collected for analysis.

##### Preparation of Samples

To remove insoluble material, samples from in vitro gastrointestinal digestions were cooled to 5 °C and centrifuged at 17,500 rpm for 10 min. After this, the supernatants were withdrawn and frozen for further analysis.

##### Total Polyphenolic Content

The total polyphenolic content (TPC) in the samples after in vitro gastrointestinal digestion was estimated using the Folin–Ciocalteu spectrophotometric test according to the method described in Section 2.4.1.

##### Antioxidant Activity

The antioxidant activity (AA) of the samples after in vitro gastrointestinal digestion was determined by the DPPH method according to the method described in Section 2.4.3. The results were expressed in mM Trolox equivalent (TE) per 100 g of yogurt (mM TE/100 g). TPC and AA of yogurt samples after in vitro gastrointestinal digestion were determined at different storage periods (1, 8, 15, 23, and 30 days).

### 2.8. Mathematical Modeling

The MATLAB program (MathWorks, Inc., Natick, MA, USA) was used for the information analysis in order to determine the influence of the concentrations of microencapsulated summer savory and rosemary extracts, and storage time on the overall acceptability, the pH values, the textural parameters, the total polyphenol content (in vitro), and the antioxidant activity (in vitro) of the yogurt samples. Names of all measured parameters are displayed in the rectangles of the graph. The mutual information values (bits) are indicated on the graph arrows. The more pronounced the influence of concentrations of summer savory and rosemary extracts and storage time on the measured parameters, the higher the bit value [30].

### 2.9. Statistical Analysis

The measurements in this study were performed in triplicate and are presented as mean values ± standard error of the mean. Calculations were performed using Microsoft Office Excel 2007 (Microsoft, Redmond, WA, USA). Staturphics, Centurion XVI 16.1.17 (Statgraphics Technologies, Inc., The Plains, VA, USA) program was used for one-way analysis of variance (ANOVA) according to Tukey’s test at a significance level of *p* ≤ 0.05.

## 3. Results and Discussion

### 3.1. Extracts Analysis

The total polyphenolic content from the summer savory extract (SE) and rosemary extract (RE) was determined using the linear equation of the standard calibration curve: y = 0.5811x + 0.0072 (R^2^ = 0.9994). The amount was expressed as mg GAE/g DW and is presented in Table 2, together with the antioxidant activity (DPPH) in the presence of plant extract. Individual polyphenolic compounds in SE and RE were identified using high-performance liquid chromatography equipped with a photodiode array detection-mass (HPLC–DAD–ESI-MS) method.

It can be observed that of the two plants, SE has a higher amount of polyphenols (43.10 mg GAE/g DW), compared to RE (38.63 mg GAE/g DW). The amount of the obtained total polyphenols were compared with results obtained by other authors. The results differ due to the type of solvent used for extraction, the extraction method used, as well as the plant. The amount of total phenolic in the aqueous extract (AQ) and non-esterified phenolic fraction from rosemary determined by Afonso et al. [31] was 16.67 mg GA/g leaf, respectively, 8.59 mg GA/g leaf. The total phenolics found by Tavassoli and Djomeh [32] was 4.99 g GA/100 g dry leaves in the extract obtained by Soxhlet with pure methanol. The amount of total phenolic contents determined in an ethanol–water (30:70) extract was 10.42 mg GA/g of dry material [33]. For summer savory, Predescu et al. [34] found 12.14 mg GA/g DW in the 60% aqueous ethanol extract. Depending on the extraction method used, the amount of total polyphenols varies between 119.28–151.54 mg GA/g DW using 96% ethanol as solvent [35]. Moreover, depending on the parts of the plant used, the amount can vary between 13.34–39.21 mg GA/g DW using 80% methanol as solvent [36].

In the present work, a total of 19 phenolic compounds were identified in SE and RE. Among the compounds identified, two main families of phenolic compounds were found: phenolic acids and flavonoids, among others.

The total phenolic content was 6.474 mg/g DW SE and 7.618 mg/g DW RE. As shown in Table 1, the major compounds quantified in the SE were methyl-rosmarinate and rosmarinic acid (3.141 mg/g DW and 1.130 mg/g DW, respectively), followed by compounds such as isorhamnetin-glucoside, luteolin-(apiosyl-malonyl)-glucoside, carnosol, cirsimarin, rosmadial, epigallocatechin, apigenin-diglucoside, definidin-(p-coumaroyl)-glucoside, cyanidin-(p-coumaroyl)-glucoside, and cyanidin-glucoside.

Among eleven phenolic acids and flavonoids identified in RE, methyl-rosmarinate was present with the highest mass fraction (2.876 mg/g DW) of phenolic acids, followed by rosmarinic acid (1.447 mg/g DW), carnosol (0.652 mg ), rosmadial (0.404 mg/g DW), and carnosic acid (0.114 mg/g DW), while cirsimarin (0.885 mg/g DW) had the highest mass fraction among flavonoids, followed by epigallocatechin, nepetin-glucoside, luteolin-acetyl-glucuronide, hispidulin-glucoside, cirsimaritin, luteolin-glucoside, and dephinidin-(p-coumaroyl)-glucoside. The results obtained in this study are in agreement with other studies [32,33,34], where methyl-rosmarinate and rosmarinic acid are reported to be the most represented phenolic acid in rosemary and summer savory.

Phenolic compounds in extracts of summer savory and rosemary (rosmarinic acid, quercetin, carnasol, luteolin, chlorogenic acid, and rutin and apigenin-glycoside) are well known for their antioxidant and antimicrobial potential [37,38]. The studied plant extracts showed high antioxidant activity, especially the rosemary extract—1216.46 mM TE/g DW.

#### In Vitro Antimicrobial Activity

The antimicrobial properties of SE and RE against gram-positive bacteria (*Bacillus cereus*, *Enterococcus faecalis*, *Staphylococcus aureus*, *Geobacillus stearothermophilus*), gram-negative bacteria (*Escherichia coli*, *Acinetobacter baumannii*, *Salmonela abony*), and yeast (*Candida albicans*), have been assessed in this study. The results shown in Table 3 indicate that the SE and RE are efficiently suppressing the growth of microorganisms with variable efficacy. SE had the maximum zone of inhibition against *Staphylococcus aureus* (26.3 mm), whereas RE showed a maximum zone of inhibition against Geobacillus stearothermophilus (27.0 mm). In the antifungal analysis, SE and RE had valuable results against Candida albicans with inhibition zone (29.3 mm and 19.0 mm), respectively. Our data confirmed that SE and RE have antibacterial and antifungal activity.

Evaluating the results obtained after performing the antimicrobial screening, it was found that both extracts had an effect both on gram-positive and gram-negative bacteria as well as on yeast of the genus *Candida.* The largest inhibition area for RE was recorded in *Geobacillus stearothermophilus* (27.0 mm) and *Staphylococcus aureus* (21.3 mm). SE was more active on the bacteria *Staphylococcus aureus* (with a diameter of the inhibition zone 26.3 mm) and *Bacillus cereus* (with a diameter of the inhibition zone 25.3 mm). The antifungal activity was more pronounced for the SE (29.3 mm) compared to the RE (19.0 mm). Both extracts demonstrated higher activity on gram-positive microorganisms compared to gram-negative ones.

Table 3 represents the MIC and MBC/MFC values of extracts against the selected bacterial and yeast species, using the broth dilution method.

By examining the growth of various microbial strains at different extract concentrations, a more precise understanding of their inhibitory effect can be gained. RE demonstrated superior activity against all microorganisms tested, exhibiting the lowest values for both MIC and MBC/MFC (from 0.7 to 2.8 and 0.7 to 11.2 mg/mL, respectively, for gram-positive bacteria; from 11.2 to 22.5 and 22.5 to 45.0 mg/mL, respectively, for gram-negative bacteria; and from 2.8 and 5.6 mg/mL, respectively, for yeast). The SE was also active on the studied microorganisms, but in higher concentrations (from 1.4 to 5.6 and 2.8 to 5.6 mg/mL, respectively, for gram-positive bacteria; from 5.6 to 45.0 and 11.2 to 45.0 mg/mL, respectively, for gram-negative bacteria; and from 5.6 and 11.2 mg/mL, respectively, for yeast).

Researchers have constantly reported the necessity to search for new antimicrobial agents active against resistant microorganisms. An alternative to antibiotics is plant extracts that are less likely to generate antimicrobial resistance due to the wide variety of active compounds [39].

The antimicrobial activity of RE and SE has been determined and demonstrated in various studies. Thus, Fernández-López et al. [40], evaluated the antibacterial activity of rosemary extracts and they found a higher antibacterial activity of them compared to other extracts studied. Only rosemary extracts were able to inhibit the 11 bacteria studied (such as *L. lactis*, *B. thermosphacta*, *L. carnosum*, *B. thermosphacta*, *L. innocua*, *L. sake*, *B. thermosphacta*, *L. mesenteroides* subsp. *mesenteroides*, *L. monocytogenes*, *L. mesenteroides* subsp. *dextranicum*, and *L. curvatus*).

The results of the current study indicate that RE and SE contain high amounts of phenols and flavonoids. Polyphenols exhibit important antimicrobial activity, the mechanisms of which have not yet been fully recognized [41]. Known mechanisms include the ability to alter the permeability of cell membranes, changes in several intracellular functions caused by binding of phenols to enzymes, or loss of cell wall integrity due to various interactions with the cell membrane [42].

Several studies have reported that plant extracts rich in polyphenols are able to inhibit the growth of spoilage bacteria and fungi and have suggested their utility in the food industry [41,43]. It is also known that phytochemicals do not work as effectively as heterogeneous extracts [44]. This is important as concerns have been reported about the increasing number of foodborne outbreaks caused by pathogens associated with antibiotic resistance [45].

Many studies have reported that gram-negative bacteria are resistant to many antibacterials, due to the hydrophilic surface of their outer membrane and associated enzymes in the periplasmic space, which are able to break down these molecules [46]. However, the results of this study showed that the tested gram-negative pathogens (*Escherichia coli*, *Salmonella abony*, and *Acinetobacte baumannii*) showed different sensitivity to the action of the studied extracts. According to certain authors, damage to the cell wall and cytoplasmic membrane may result in the loss of structural integrity and a reduction in the membrane’s ability to serve as a permeability barrier due to damage to the cell wall and cytoplasmic membrane [47]. The change in cell structure could destabilize the cell membrane and increase its fluidity, leading to increased permeability and leakage of various vital intracellular constituents [46,48]. Moreover, it has been shown that the use of polyphenolic extracts can exert a double positive effect, the simultaneous inhibition of pathogens, and the stimulation of beneficial bacteria [7].

### 3.2. Beads Characterization

#### 3.2.1. SEM Analysis

**Figure 1:**
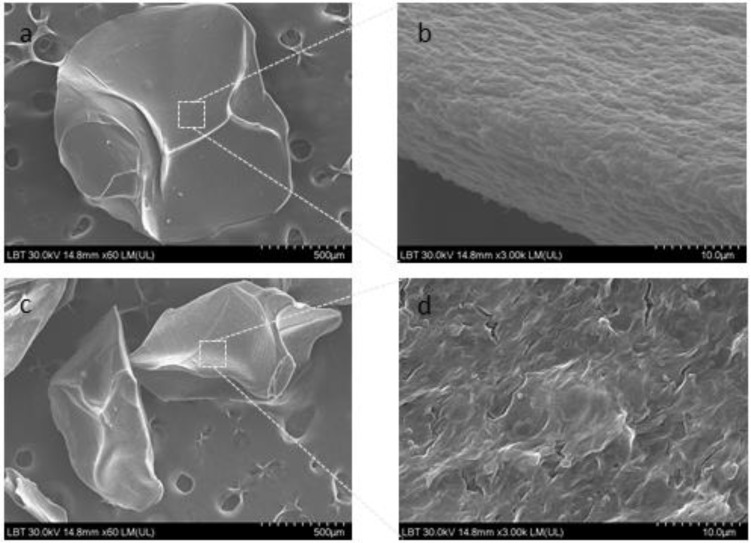
SEM micrographs of the microencapsulated samples; (**a**,**b**) MSE, (**c**,**d**) MRE.

The morphological characteristics of the beads were assayed through SEM (Figure 1). The surface of the beads appears smooth at low magnifications (Figure 1a,c) and without pores, as seen at high magnifications (Figure 1b,d).

SEM analysis showed a similar morphology of the surface of the samples. Thus, the two samples, MRE and MSE, have a smoother surface. Other studies had similar results when the samples were investigated through SEM [49,50,51].

The size of microcapsules seems to be around 1 mm. It is not possible to obtain more details due to measurements in a vacuum atmosphere.

#### 3.2.2. FTIR Analysis

**Figure 2:**
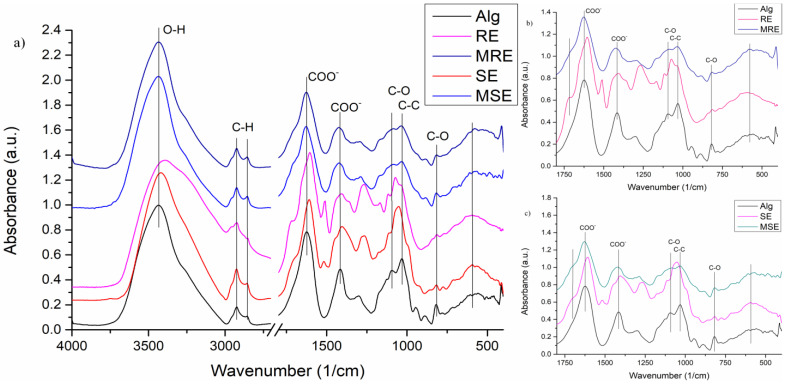
The FTIR spectra of (**a**) Na alginate, rosemary, and summer savory extract, and the encapsulated extracts, 4000–400 cm^−1^ spectral domain, 2750–1850 cm^−1^ splitted; (**b**) Na alginate, rosemary extract, and the encapsulated extract, 1800–400 cm^−1^ spectral domain; (**c**) Na alginate, summer savory extract and the encapsulated extract, 1800–400 cm^−1^ spectral domain.

The characteristic absorption peaks of Na alginate (Figure 2) can be assigned as follow: 3434 cm^−1^ (stretching vibrations of -OH groups), 2924 and 2855 cm^−1^ (asym. and sym. stretching peaks of CH_2_ groups), 1624 and 1416 cm^−1^ (asym. and sym. stretching peaks of COO- salt groups), 1301 cm^−1^ (C-O stretching), 1173 and 1124 cm^−1^ (C-C stretching), 1095 and 1031 cm^−1^ (stretching of groups C-O and C-O-C in mannuronic, and guluronic units, respectively) [52], 946 cm^−1^ (C-O stretching of pyranosyl ring and the C-O stretching with contributions from C-C-H and C-O-H deformation), 818 cm^−1^ (C-O vibration of groups in α-configuration of the glucuronic units) [53].

The FTIR spectra of rosemary, RE, and summer savory, SE (Figure 2) are very similar. The characteristic vibration bands that can be found in the spectra of the two extracts are the following: a strong broad absorption band around 3400 cm^−1^ (phenolic -OH and -NH), 2924 and 2855 cm^−1^ (alkane -C-H), 1716 cm^−1^ (carbonyl -C=O), 1605 cm^−1^ (-NH, H-O-H, and alkene -C=C-) [54,55], 1510 cm^−1^ (aromatic -C=C-), 1407 cm^−1^ (alkane -C-H), 1360sh cm^−1^ (RE) (alkane -C-H) [56], 1266 cm^−1^ (ether C-O-C), 1167 and 1116 cm^−1^ (alcohol -C-O), 815 cm^−1^ (carboxylic O-C=O), and 630 cm^−1^ (C-Cl). Few differences can be observed between the two spectra, namely: the bands in 1716, 1510, 1167, and 1116 cm^−1^, respectively, show lower intensity in the spectrum of the savory extract.

The FTIR spectra of the RE or SE encapsulated in the Alg, MRE, and MSE, show wider absorption bands than starting compounds, but the characteristic vibrational bands of components can be found in the spectra, slightly shifted.

In the case of MRE (Figure 2) the following significant changes were observed: the -C=O stretching is shifted from 1716 cm^−1^ to 1706 cm^−1^, the -C=C- of extract and -COO- vibration of alginate shifted from 1615 cm^−1^ and 1605 cm^−1^, respectively, to 1626 cm^−1^. The vibrational band of RE from 1510 cm^−1^ disappear, the alkane -CH stretching of RE from 1407 cm^−1^ and COO- stretching of alginate from 1416 cm^−1^ shifted to 1424 cm^−1^, the ether -C-O-C vibration from 1266 cm^−1^ shifted to 1292 cm^−1^, and the -C-O stretching of RE from 1072 cm^−1^ shifted to 1084 cm^−1^.

In the spectrum of MSE (Figure 2), the following differences were observed compared to the spectra of the starting substances: the -C=O stretching is shifted from 1701 cm^−1^ to 1709 cm^−1^, the -C=C- of extract and -COO- vibration of alginate shifted from 1608 cm^−1^ and 1624 cm^−1^, respectively, to 1627 cm^−1^. The vibrational band of SE from 1517 cm^−1^ disappears, the alkane -CH stretching of RE from 1404 cm^−1^ and COO- stretching of alginate from 1416 cm^−1^ shifted to 1422 cm^−1^, the ether -C-O-C vibration from 1266 cm^−1^ shifted to 1286 cm^−1^, and the -C-O stretching of SE from 1052 cm^−1^ shifted to 1032 cm^−1^.

The identified changes in the MRE and MSE spectra compared to the spectra of components, Alg and RE or SE, respectively, can be attributed to the existence of weak physical interactions between the components.

#### 3.2.3. Encapsulation Efficiency of Summer Savory and Rosemary Extracts in Alginate Beads

The efficiency of encapsulation for both plants was 4.79% for summer savory and 14.76% for rosemary. Similar encapsulation efficiencies, between 11 and 18%, were obtained for longan seed extract incorporated in the alginate/chitosan beads [57].

### 3.3. Concentrated Yogurt with Microencapsulated Plant Extracts Characterization

#### 3.3.1. Physicochemical Analysis of the Concentrated Yogurt with Microencapsulated Plant Extracts

The physicochemical parameters of the concentrated yogurt samples (total dry matter content, protein, and fat content) evolved non-essentially as a function of the concentration of microcapsules, either using thyme or rosemary. The 0.60% CYMSE sample presented a slightly lower protein and fat content (5.67 ± 0.01% and 8.97 ± 0.02%, respectively) compared to plain yogurt (5.69 ± 0.03% and 9.02 ± 0.02%, respectively). Regarding the total dry matter content, the samples with added MSE had a higher dry matter content 19.51 ± 0.03% (0.60% YMSE) in contrast to plain yogurt (19.05 ± 0.01%). Data taken from the literature revealed a similar correlation [58,59,60]. 

#### 3.3.2. Evolution of the Concentrated Yogurt with Microencapsulated Plant Extracts Characteristics during Storage

The analysis of sensory properties using the human senses is a very useful tool in food characterization. Appearance and consistency, color, odor, taste as well as overall acceptance were evaluated by 15 assessors of the sensory panel using a 5-point scoring scale and the results are presented in Table S1.

On the first day of storage, all yogurt samples showed high overall acceptability scores. However, the use of microencapsulated plant extracts in the fortification of concentrated yogurt at a concentration of 0.60% determined the appearance of a plant residue that intensifies during storage which led to the decrease of the taste score up to 3.40 in the case of the addition of MSE and up to at 3.46 in the case of the addition of MRE.

Starting with the 23rd day of storage, an overall acceptance deterioration of the CY sample was found from 4.75 points on the 23rd day of storage, to 4.16 points on the 30th day of storage. The deterioration of the sensory quality of this sample was determined by the separation of the whey at the surface of the curd, accompanied by the appearance of the slightly sour taste of the yogurt. This behavior is due to the decrease in the pH of the CY sample during storage (Table 4), which may have a contraction effect in the casein micellar matrix causing greater whey removal [61]. 

With the addition of 0.30% and 0.45% microencapsulated plant extracts in concentrated yogurt, no changes were found in the sensory quality during the 30 days of storage, both in the case of using MSE and MRE. The addition of microencapsulated plant extracts, at concentrations higher than 0.3%, had a positive impact on the storage stability of concentrated yogurt compared to CY. This proves that the structure of microcapsules can tolerate an acidic environment and promotes the controlled release of polyphenolic compounds [14], with proven antioxidant and antimicrobial effects. In addition, the sodium alginate in the structure of the microcapsules favored the retention of whey, thus contributing to the mesh effect in the three-dimensional network of the gel formed in yogurt [62].

Data from the literature is contradictory, in the study by de Moura et al. [58] it was demonstrated that yogurt samples with hibiscus extract encapsulated were highly appreciated by the group of evaluators. The use of natural bioactive compounds encapsulated from red pepper waste in yogurt led to higher overall acceptability scores compared to the control sample [60]. Additionally, research on the effect of the fortification of seated yogurt enriched with microcapsules containing omega-3 fatty acids showed a slight decrease in the acceptability of the product [62].

The pH values of concentrated yogurt with microencapsulated plant extracts, during the 28-day storage period at 4 °C, are shown in Table 4. All yogurt samples showed a decrease in pH during storage. In the case of plain yogurt, these values were 4.59 on day 1, 4.38 on day 8, and 4.20 on day 30. The addition of MSE to yogurt led to a decrease in the pH value from 4.59 in the case of 0.15%CYMSE to 4.51 in the case of 0.6% CYMSE. Similar data were also obtained in the case of yogurt samples with the addition of MRE. The slight decrease in pH in yogurt samples, with the addition of microencapsulated plant extracts, occurs as a result of the gradual release of phenolic acids from the extracts, a fact also confirmed by the research carried out by Azarashkan et al. [63].

During the storage period, the pH of the CY sample and concentrated yogurt with microencapsulated plant extracts decreased gradually. Although, on the first day of storage, the pH of the CY sample was higher than the samples of yogurt with microencapsulated plant extracts, at the end of the storage, the pH was lower than in the rest of the samples. On the 30th day of storage, the pH values of the concentrated yogurt varied between 4.20 (CY), 4.45 (0.6%CYMSE), and 4.44 (0.6%CYMRE). The decrease in pH over time is the result of the post-acidification of the products, related to the continuation of the fermentation process by the lactic bacteria present in the yogurt during storage [64].

The addition of microencapsulated plant extracts to yogurt led to the inhibition of the post-fermentation process during storage. According to data from the literature, the activity of different extracts with antibacterial properties can be limited by the pH value of the fortified products [65]. Therefore, the preservation of the active compounds in encapsulated form is essential to maintain their stability and effectiveness until the consumption of the enriched product [66]. The results presented in this study are also supported by other researchers [58,67,68].

Knowing the textural properties of yogurts is important from a technological point of view and determines the purchasing power of food. The textural properties of fermented dairy products depend on their structural arrangement and the microstructure of the protein network [27]. The evolution of the TPA parameters (hardness, cohesiveness, adhesiveness, and gumminess) of the concentrated yogurt with microencapsulated plant extracts during the 30-day storage at 4 °C are shown in Table 5.

The hardness of yogurt samples was reduced with increasing MSE concentration from 38.31g (CY) to 31.54g(0.6% CYMSE) and 29.27g (0.6% CYMRE). This decrease is due to the weakening of the protein network in the yogurt matrix or a slight decrease in the pH value, as indicated in [69]. The variance of the other textural parameters had the same tendency as the hardness. Thus, the values of adhesiveness and gumminess were reduced with the increase in the amount of microencapsulated plant extracts. Mean cohesiveness values were lower for yogurt samples with MSE and MRE than for plain yogurt, this may be due to the reduced strength of protein-protein bonds [59]. According to the results obtained by Hashim et al. [70] the addition of bioactive compounds extracted from date palm seeds (*Phoenix dactylifera* L.) had a slight effect on the textural profile of the yogurt compared to the control.

However, for 30 days the textural parameters (hardness, adhesiveness, and gumminess) of the fortified concentrated yogurt samples increased, and in plain yogurt, there was a decrease in these values, which suggests that the addition of microencapsulated plant extracts has led to a stable system and the formation of a strong three-dimensional network in the yogurt. In the case of the 0.60% CYMSE sample, hardness and gumminess increased from 31.54 g to 35.05 g and from 2.37% to 2.94%, respectively. Additionally, the adhesiveness decreased from 35.24 g⋅s to 33.55 g⋅s. Similar results were reported by other authors [67,71,72].

Therefore, MRE and MSE caused the degree of crosslinking in the gel network to increase, and as a result, a firmer gel structure was formed. Such an effect could be attributed to the better water retention capacity in concentrated yogurt samples with microencapsulated plant extracts compared to plain yogurt.

Recent studies are focused on the fortification of dairy products with phenolic compounds in free form (extract, powder, etc.) as well as in the form of micro/nanocapsules [7]. According to the studies carried out by Trigueros et al. [73], the stability of phenolic and color compounds in yogurt fortified with pomegranate juice is influenced by storage temperature, pH, the chemical composition of yogurt, especially fat, as well as the content and type of phenolic compounds. It was established that these parameters condition the formation of reversible or irreversible bonds between milk proteins and phenolic compounds in pomegranate juice. The establishment of interactions between milk proteins and phenolic compounds was also found in the case of obtaining yogurt with the addition of encapsulated and non-encapsulated grape seed extract [67]. The loss of phenolic compounds from the yogurt obtained by incorporating the unencapsulated grape seed extract during storage (21 days) under refrigerated conditions was 46%.

The results of these studies support the fact that in order to exert their activity at a systemic level, phenolic compounds from plants must be bioavailable, that is, they must be released from the food matrix, absorbed at the intestinal level, and reach the target organs [74]. The presence of milk proteins, which can bind and precipitate phenolic compounds released from plant extracts, may be responsible for this decrease in total phenolic compounds in yogurt fortified with free extracts. The polypeptide chain of milk proteins is characterized by the presence of a large number of hydrophobic regions, which facilitate the formation of bonds with biochemical compounds with different degrees of affinity, including phenolic compounds [75,76]. The size of the encapsulated particles also influences the stability of the polyphenolic compounds, with larger microcapsules providing better protection than smaller microcapsules [77]. Therefore, encapsulation of phenolic compounds in plant extracts would increase their bioavailability. In the given study, yogurt samples with the addition of microencapsulated plant extracts were subjected to an in vitro gastrointestinal digestion protocol, after which the total polyphenolic content (TPC) and antioxidant activity (AA) of the yogurt samples during storage were determined (Table 6).

As shown in Table 6, the TPC extracted in concentrated yogurt samples with the addition of MSE and MRE after in vitro digestion was influenced by the type of encapsulated extract and the concentration of microencapsulated plant extract. In the case of yogurt samples with the addition of MSE, there was an increase in the extracted TPC from 5.24 mg GAE/100 g yogurt (0.15% CYMSE) to 20.25 mg GAE/100 g yogurt (0.60% CYMSE), which had resulted in a 0.86% increase in AA. Similarly, increasing the concentration of MRE addition in yogurt from 0.15% to 0.60% led to an increase in the extracted TPC from 4.95 mg GAE/100 g to 19.82 mg GAE/100 g yogurt, which resulted in an increase of 1.05 times the AA. A high TPC was observed in yogurt added with beet extract encapsulated with maltodextrin and inulin (8.288 mg GAE/g and 7.352 mg GAE/g respectively) [64], and in yogurt added with cactus pear (*Opuntia ficus indica*) (14.67 mg GAE/g) [78].

During the 30 days of storage, TPC released in concentrated yogurt with microencapsulated plant extracts registered a constant increase. The retention of phenolic compounds during storage was 126.02% for the 0.60% CYMSE and 124.35% for the 0.60% CYMRE. Similar results were obtained for the other fortified yogurt samples. Šeregelj et al. [60] reported that the retention of polyphenols in yogurt with encapsulated natural bioactive compounds from red pepper waste increased during storage for the control yogurt (up to 115.48%) and for the fortified yogurt (up to 123.73%). The same trend was reported by Saponjac et al. [79], whereby the retention of polyphenols in cookies also increased during storage, suggesting that the degradation of conjugated polyphenols releases free hydroxyl groups that determined the increase in the Folin–Ciocalteu test results. Contrary results were reported by Flores-Mancha et al. [64] which are characterized by a decrease in the content of polyphenols in yogurt added with beet extract encapsulated with maltodextrin and inulin during storage. The storage of foods with high water activity probably facilitates oxidation reactions, causing phenolic compounds that store oxygen and produce an enriched environment with strong radical scavenging and reducing properties [68].

Therefore, sodium alginate encapsulation of SE and RE was effective in stabilizing polyphenolic compounds over the 30 days of storage.

Throughout the storage period of yogurt samples with MSE and MRE, the AA increased with the rise in the content of microencapsulated plant extracts, and the highest antioxidant activity was observed in 0.60% CYMSE and 0.60% CYMRE after 30 days (0.392 ± 0.017 mmol TE /100 g and, respectively, 0.432 ± 0.009 mmol TE/100 g). While plain yogurt samples showed a decrease in AA from 0.122 mM TE/100 g on the first day of storage to 0.101 mM TE/100 g on the 30th day of storage. The release of antioxidant peptides and amino acids, encoded in milk protein sequences, during in vitro digestion may explain the AA value in plain yogurt. Furthermore, casein peptides with high levels of histidine, tryptophan, methionine, and tyrosine have been previously shown to be released during in vitro digestion of milk or dairy products, exhibiting antioxidant abilities [8]. According to research carried out by Pitalua et al. [68] during storage, there was a decrease in AA, followed by a period of stability for 10 days and an increase in AA until day 14. This behavior could be due to polyphenols with high antioxidant activity that were formed or released during the first days of storage due to the adsorption of water on the surface of the microcapsules or the interaction of some components of the stored sample with oxygen or with other components of the sample [80,81]. The AA of yogurt with doum powder encapsulated in liposomes and yogurt enriched by olive leaf phenolics within nanoliposomes increased or maintained during storage compared to the non-encapsulated form of the extracts [72,82].

The addition of 0.30–0.45% microencapsulated plant extracts in the concentrated yogurt determined the inhibition of the post-fermentation process, the improvement of the textural parameters of the yogurt during storage, thus the shelf life of the yogurt increased by 7 days, compared to plain yogurt.

The yogurt sample with the addition of 0.9% MSE and 0.9% MRE demonstrated a significant AA and high texture parameters, however, from a sensory point of view, it was rated with a low score due to the pronounced herbal taste. The addition of 0.15% microencapsulated plant extracts in the yogurt was insufficient to ensure the stability of the yogurt during the 30 days of storage.

### 3.4. Mathematical Modeling

**Figure 3:**
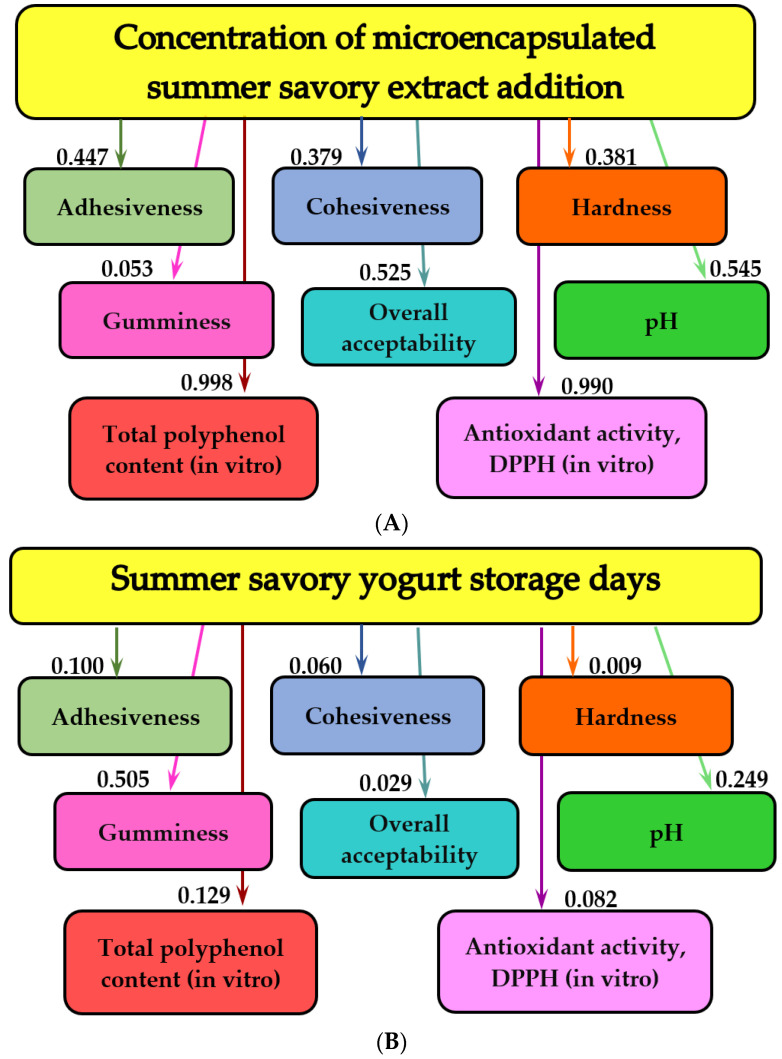
The informational analysis of the concentrations of MSE added (**A**) and storage days (**B**) on the overall acceptability, pH, textural parameters, the total polyphenol content (in vitro), and the antioxidant activity (in vitro) of concentrated yogurt samples.

The measure of the influence of both the concentrations of MSE and MRE added, and the storage period of the concentrated yogurt samples, on the overall acceptability, pH values, texture parameters, total polyphenol content (in vitro), and the antioxidant activity (in vitro), were evaluated by analysis of mutual information. Figure 3 demonstrates the mutual analysis of the influence of concentrations of MSE added (Figure 3A) and storage days (Figure 3B) on the overall acceptability, pH, textural parameters (adhesiveness, cohesiveness, hardness, and gumminess), the total polyphenol content (in vitro), and the antioxidant activity (in vitro) of concentrated yogurt samples.

Figure 3A shows that the concentration of MSE added to the concentrated yogurt samples essentially influences TPC (mutual information 0.998 bits) and AA (0.990 bits). These are followed, in descending order, by the pH values (by 0.545 bits), overall acceptability (0.525 bits), adhesiveness (0.447 bits), and cohesiveness (0.379 bits). The lowest effect was exhibited on the gumminess (0.053 bits).

In the case of the storage time of concentrated yogurt samples with MSE (Figure 3B), gumminess has the most influence (0.505 bits). Following, in descending order of influence, are pH (0.249 bits) and TPC (0.129 bits) values. The lowest effects were exhibited on the other textural parameters, the antioxidant activity (0.082), and the overall acceptability (0.029 bits).

**Figure 4:**
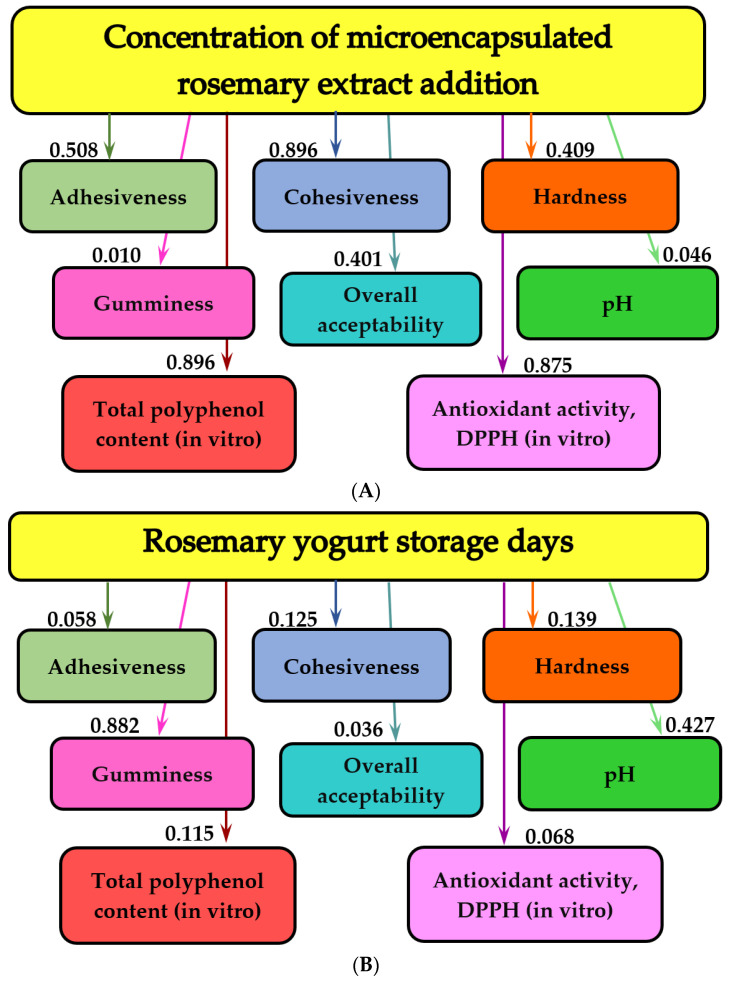
The informational analysis of the concentrations of MRE added (**A**) and storage days (**B**) on the overall acceptability, pH, textural parameters, the total polyphenol content (in vitro), and the antioxidant activity (in vitro) of concentrated yogurt samples.

Figure 4 presents the mutual information analysis of the influence of added MRE concentrations (Figure 4A) and storage days (Figure 4B) on the same parameters as in the case of MSE yogurts samples. 

As shown in Figure 4A, the concentrations of MRE added to yogurt samples equally influence the cohesiveness and TPC (mutual information 0.896 bits) the most. Following, in descending order of influence, are AA (0.875 bits), adhesiveness (0.508 bits), hardness (0.409 bits), and overall acceptability (0.401 bits). The pH values and the gumminess are insignificant in influence, 0.046 and 0.010 bits, respectively.

Figure 4B presents the results of the information analysis and shows that storage days of yogurt samples have the most influence on gumminess (0.882 bits) and pH values (0.427 bits). These are followed, in descending order, by hardness (by 0.139 bits), cohesiveness (0.125 bits), and TPC (0.115 bits). The lowest effects were exhibited on the antioxidant activity AA (0.068 bits), adhesiveness (0.058 bits), and overall acceptability (0.036 bits).

Mutual information analysis was applied in the study of the influence of vegetable addition and storage time on the quality of yogurt, for 20 days [27] and gingerbreads, for 45 days [83]. The effects of storage time on the quality of beef dry-aging, for 35 days [84] and various quantities of sea buckthorn berry flour on wheat bread were investigated [85].

## 4. Conclusions

The antioxidant and antimicrobial activity of extracts from two aromatic plants - *Satureja hortensis* L. and *Rosmarinus officinalis* L. are of interest in the development of functional products with an extended shelf life. The major compounds identified in the SE were methyl-rosmarinate and rosmarinic acid, isorhamnetin-glucoside, luteolin-(apiosyl-malonyl)-glucoside, carnosol, cirsimarin, rosmadial, epigallocatechin, apigenin-diglucoside, definidin-(*p*-coumaroyl)-glucoside, cyanidin-*(p*-coumaroyl)-glucoside, and cyanidin-glucoside. The extracts had an effect on both gram-positive and gram-negative bacteria, as well as on *Candida* yeast. Sodium alginate encapsulation of SE and RE led to the stabilization of polyphenolic compounds during 30 days of storage.

Yogurt fortified with MSE and MRE showed a higher content of re-released polyphenols under conditions of in vitro gastrointestinal digestion compared to plain yogurt. Therefore, the manufactured MSE and MRE-enriched yogurt can combine the biological activities of yogurt components with those of the phenolic compounds of thyme and rosemary extracts and can be considered a functional food with improved health properties.

The industrial production of concentrated yogurt fortified with encapsulated plant extracts will lead to the diversification of the assortment of fermented dairy products with health benefits, increasing the shelf life of yogurt. In addition, microencapsulation diminishes the negative effect of plant extracts on the sensory characteristics of foods.

Further research could aim to develop more complex delivery systems that would allow the fortification of fermented dairy products simultaneously with multiple bioactive compounds. In some cases, co-encapsulation can be complicated due to the different and not always compatible physicochemical properties of the bioactive ingredients.

## Figures and Tables

**Table 1:** Polyphenols used as standards in HPLC analysis of summer savory and rosemary extract.

Compound	Max Absorption, nm	Retention Time, min	*m*/*z* [M+H]^+^	Polyphenol Classes
Cyanidin-glucoside	520, 280	12.14	449	Antocianin
Epigallocatechin	280	13.17	306	Flavanol
Apigenin-diglucoside	340, 270	13.46	595	Flavone
Luteolin-glucoside	350, 260	16.14	449	Flavone
Dephinidin-(p-coumaroyl)-glucoside	523, 330, 280	15.91	611	Anthocyanin
Nepetin-glucoside	350, 265	16.83	479	Flavone
Cyanidin-(p-coumaroyl)-glucoside	520, 330, 280	17.15	595	Anthocyanin
Luteolin-glucuronide	350, 260	17.83	463	Flavone
Hispidulin-glucoside	340, 260	18.13	463	Flavone
Luteolin-(apiosyl-malonyl)-glucoside	350, 260	18.54	667	Flavone
Isorhamnetin-glucoside	350, 255	18.87	478	Flavonol
Luteolin-acetyl-glucuronide	350, 260	18.98	505	Flavone
Rosmarinic acid	330	20.13	360	Hydroxycinnamic acid
Cirsimarin	340, 270	20.56	477	Flavone
Methyl-rosmarinate	330	22.39	375	Hydroxycinnamic acid
Carnosol	330, 270	23.17	331	Phenolic terpene
Rosmadial	330	23.56	345	Phenolic terpene
Cirsimaritin	330, 260	24.39	315	Flavone
Carnosic acid	270	25.49	332	Phenolic terpene
Not identified	270	24.85	624, 249	

**Table 2:** The content of total and individual polyphenols and the antioxidant activity of summer savory extract and rosemary extract used for experiments.

Indices	Quantity	
	Summer Savory Extract	Rosemary Extract
**Total Polyphenol content (Folin–Ciocalteu),** mg GAE/g DW	43.10 ± 0.29	38.63 ± 0.29
**Total Individual polyphenols,** mg/g DW	6.474 ± 0.005	7.618 ± 0.006
Cyanidin-glucoside	0.016 ± 0.002	-
Epigallocatechin	0.082 ± 0.001	0.411 ± 0.009
Apigenin-diglucoside	0.036 ± 0.002	-
Luteolin-glucoside	-	0.065 ± 0.003
Dephinidin-(p-coumaroyl)-glucoside	-	0.031 ± 0.002
Nepetin-glucoside	-	0.238 ± 0.012
Cyanidin-(p-coumaroyl)-glucoside	0.030 ± 0.002	-
Luteolin-glucuronide	-	0.047 ± 0.003
Hispidulin-glucoside	-	0.193 ± 0.002
Luteolin-(apiosyl-malonyl)-glucoside	0.445 ± 0.013	-
Isorhamnetin-glucoside	0.614 ± 0.007	-
Luteolin-acetyl-glucuronide	-	0.201 ± 0.002
Rosmarinic acid	1.130 ± 0.11	1.447 ± 0.09
Cirsimarin	0.259 ± 0.04	0.885 ± 0.03
Methyl-rosmarinate	3.141 ± 0.02	2.876 ± 0.02
Carnosol	0.263 ± 0.01	0.652 ± 0.13
Rosmadial	0.233 ± 0.04	0.404 ± 0.08
Cirsimaritin	-	0.054 ± 0.02
Carnosic acid	-	0.114 ± 0.03
Not identified	0.225 ± 0.02	-
**DPPH Antioxidant activity,** mM TE/g DW	**580.16 ± 1.83**	**1216.46 ± 2.42**

The results are presented as the mean of three measurements ± SD (standard deviation).

**Table 3:** Antimicrobial activity of the plant extracts against bacteria and yeast strains.

Test Strains	Zone of Inhibition, mm *		MIC, mg/mL		MBC/MFC, mg/mL	
	RE	SE	RE	SE	RE	SE
**Gram-positive bacteria**						
*Bacillus cereus*	18.3 ± 0.5 ^d,e^	25.3 ± 0.6 ^f^	0.7 ± 0.1 ^a^	2.8 ± 0.1 ^a^	0.7 ± 0.1 ^a^	5.6 ± 0.2 ^b^
*Enterococcus faecalis*	18.7 ± 0.3 ^d,e^	13.7 ± 0.7 ^c^	2.8 ± 0.1 ^b^	5.6 ± 0.1 ^b^	11.2 ± 0.5 ^c^	5.6 ± 0.1 ^b^
*Staphylococcus aureus*	21.3 ± 0.2 ^e^	26.3 ± 0.6 ^f^	1.4 ± 0.2 ^a^	1.4 ± 0.1 ^a^	11.2 ± 0.4 ^c^	5.6 ± 0.3 ^b^
*Geobacillus stearothermophilus*	27.0 ± 0.6 ^f^	20.0 ± 0.5 ^e^	0.7 ± 0.1 ^a^	1.4 ± 0.2 ^a^	1.4 ± 0.1 ^a^	2.8 ± 0.1 ^b^
**Gram-negative bacteria**						
*Escherichia coli*	15.0 ± 0.6 ^c,d^	10.0 ± 0.7 ^b^	22.5 ± 0.5 ^d^	22.5 ± 0.6 ^d^	45.0 ± 0.7 ^e^	22.5 ± 0.6 ^d^
*Acinetobacter baumannii*	17.0 ± 0.5 ^d^	13.0 ± 0.8 ^c^	11.2 ± 0.5 ^c^	5.6 ± 0.1 ^b^	22.5 ± 0.6 ^d^	11.2 ± 0.5 ^c^
*Salmonela abony*	13.0 ± 0.5 ^c^	8.0 ± 0.7 ^a^	22.5 ± 0.7 ^d^	45.0 ± 0.8 ^e^	22.5 ± 0.5 ^d^	45.0 ± 0.8 ^e^
**Yeast**						
*Candida albicans*	19.0 ± 0.4 ^e^	29.3 ± 0.6 ^g^	2.8 ± 0.2 ^b^	5.6 ± 0.1 ^b^	5.6 ± 0.3 ^b^	11.2 ± 0.5 ^c^

* Diameter of inhibition zone; MIC—minimum inhibitory concentration; MBC—minimum bactericidal concentration; MFC—minimum fungicidal concentration. The results are presented as the mean of three measurements ± SD (standard deviation). Different letters (^a–g^) designate statistically different results (*p* ≤ 0.05).

**Table 4:** pH value evolution of concentrated yogurt with microencapsulated plant extracts during storage.

Storage Period, Days	Samples								
	CY	0.15% CYMSE	0.30% CYMSE	0.45% CYMSE	0.60% CYMSE	0.15% CYMRE	0.30% CYMRE	0.45% CYMRE	0.60% CYMRE
1	4.59 ± 0.0 ^k^	4.59 ± 0.0 ^k^	4.56 ± 0.01 ^j,k^	4.52 ± 0.01 ^i,j^	4.51 ± 0.01 ^i,j^	4.59 ± 0.0 ^k^	4.57 ± 0.01 ^k^	4.54 ± 0.01 ^j^	4.53 ± 0.01 ^i,j^
8	4.38 ± 0.02 ^e,f^	4.40 ± 0.01 ^f^	4.42 ± 0.02 ^f,g^	4.45 ± 0.01 ^g,h^	4.48 ± 0.01 ^h,i^	4.43 ± 0.01 ^g^	4.45 ± 0.01 ^g,h^	4.49 ± 0.01 ^h,i^	4.51 ± 0.01 ^i^
15	4.35 ± 0.02 ^d,e^	4.37 ± 0.02 ^e,f^	4.41 ± 0.01 ^f,g^	4.44 ± 0.02 ^g,h^	4.46 ± 0.01 ^g,h^	4.43 ± 0.01 ^g^	4.45 ± 0.01 ^g,h^	4.47 ± 0.01 ^h^	4.49 ± 0.01 ^h,i^
23	4.23 ± 0.01 ^b^	4.34 ± 0.01 ^d,e^	4.40 ± 0.01 ^f^	4.43 ± 0.01 ^g^	4.45 ± 0.01 ^g,h^	4.39 ± 0.02 ^e,f^	4.40 ± 0.01 ^f^	4.45 ± 0.01 ^g,h^	4.43 ± 0.01 ^g^
30	4.20 ± 0.02 ^a,b^	4.31 ± 0.01 ^c,d^	4.38 ± 0.02 ^e,f^	4.42 ± 0.02 ^g^	4.45 ± 0.01 ^g,h^	4.32 ± 0.01 ^c,d^	4.39 ± 0.02 ^f^	4.42 ± 0.02 ^f,g^	4.44 ± 0.02 ^g,h^

The results are presented as the mean of three measurements ± SD (standard deviation). Different letters (^a–k^) designate statistically different results (*p* ≤ 0.05).

**Table 5:** Texture parameters’ evolution of concentrated yogurt with microencapsulated plant extracts during storage.

Texture Parameters	Storage Period, Day	Samples								
		CY	0.15% CYMSE	0.30% CYMSE	0.45% CYMSE	0.60% CYMSE	0.15% CYMRE	0.30% CYMRE	0.45% CYMRE	0.60% CYMRE
Hardness, g	1	38.31 ± 0.25 ^n^	35.67 ± 0.17 ^j,k^	35.15 ± 0.21 ^i,j^	33.94 ± 0.19 ^h^	31.54 ± 0.22 ^d,e^	32.41 ± 0.15 ^f^	31.79 ± 0.23 ^e^	29.60 ± 0.18 ^a,b^	29.27 ± 0.13 ^a^
	8	38.48 ± 0.31 ^n,o^	37.24 ± 0.19 ^l,m^	35.59 ± 0.20 ^j,k^	34.39 ± 0.22 ^h,i^	32.42 ± 0.16 ^f^	33.88 ± 0.21 ^h^	32.69 ± 0.18 ^f^	30.83 ± 0.22 ^c,d^	30.50 ± 0.18 ^c^
	15	38.79 ± 0.36 ^n,o^	37.76 ± 0.21 ^m,n^	35.98 ± 0.16 ^k^	34.65 ± 0.17 ^i^	33.43 ± 0.15 ^h^	34.71 ± 0.13 ^i^	33.51 ± 0.21 ^g,h^	32.20 ± 0.16 ^e,f^	31.48 ± 0.20 ^d,e^
	23	38.28 ± 0.19 ^n^	38.48 ± 0.16 ^n,o^	36.93 ± 0.22 ^l^	35.67 ± 0.25 ^j,k^	34.11 ± 0.20 ^h^	36.16 ± 0.17 ^k^	34.75 ± 0.16 ^i^	33.97 ± 0.25 ^h^	32.24 ± 0.17 ^e,f^
	30	34.19 ± 0.28 ^h,i^	35.58 ± 0.12 ^j^	37.76 ± 0.16 ^m,n^	36.92 ± 0.18 ^l^	35.05 ± 0.19 ^i,j^	37.05 ± 0.18 ^i,m^	35.97 ± 0.23 ^j,k^	34.74 ± 0.18 ^i^	33.56 ± 0.15 ^g,h^
Cohesiveness, %	1	0.070 ± 0.001 ^b,c^	0.072 ± 0.001 ^c^	0.072 ± 0.001 ^c^	0.073 ± 0.001 ^c,d^	0.075 ± 0.001 ^d,e^	0.076 ± 0.001 ^d,e^	0.077 ± 0.001 ^d,e^	0.079 ± 0.001 ^e,f^	0.080 ± 0.001 ^e,f^
	8	0.071 ± 0.001 ^b,c^	0.072 ± 0.001 ^c^	0.073 ± 0.001 ^c,d^	0.074 ± 0.001 ^c,d^	0.076 ± 0.001 ^d,e^	0.078 ± 0.001 ^e,f^	0.083 ± 0.001 ^f,g^	0.085 ± 0.001 ^g,h^	0.092 ± 0.001 ^i^
	15	0.072 ± 0.001 ^c^	0.073 ± 0.001 ^c,d^	0.077 ± 0.001 ^d,e^	0.080 ± 0.001 ^f^	0.082 ± 0.001 ^f,g^	0.078 ± 0.001 ^e,f^	0.083 ± 0.001 ^f,g^	0.085 ± 0.001 ^g,h^	0.092 ± 0.001 ^i^
	23	0.071 ± 0.001 ^b,c^	0.071 ± 0.001 ^b,c^	0.078 ± 0.001 ^e^	0.081 ± 0.001 ^f,g^	0.083 ± 0.001 ^f,g^	0.082 ± 0.001 ^f,g^	0.084 ± 0.001 ^g,h^	0.086 ± 0.001 ^h^	0.094 ± 0.001 ^i,j^
	30	0.058 ± 0.001 ^a^	0.070 ± 0.001 ^b,c^	0.079 ± 0.001 ^e,f^	0.082 ± 0.001 ^f,g^	0.084 ± 0.001 ^g,h^	0.083 ± 0.001 ^f,g^	0.085 ± 0.001 ^g,h^	0.087 ± 0.001 ^h^	0.095 ± 0.001 ^j^
Adhesiveness, g⋅s	1	39.88 ± 0.22 ^k^	37.73 ± 0.15 ^i^	37.16 ± 0.16 ^h,i^	36.26 ± 0.18 ^f,g^	35.24 ± 0.21 ^e^	37.17 ± 0.19 ^h,i^	36.35 ± 0.16 ^g^	35.35 ± 0.15 ^e^	34.96 ± 0.18 ^d,e^
	8	38.80 ± 0.17 ^j^	37.61 ± 0.18 ^i^	36.97 ± 0.22 ^h^	35.61 ± 0.20 ^e,f^	34.37 ± 0.15 ^c,d^	36.76 ± 0.15 ^g,h^	35.81 ± 0.14 ^f^	35.04 ± 0.19 ^d,e^	34.45 ± 0.17 ^c,d^
	15	37.64 ± 0.19 ^i^	37.22 ± 0.21 ^h,i^	36.54 ± 0.15 ^g^	34.82 ± 0.17 ^d,e^	33.95 ± 0.18 ^c^	35.99 ± 0.20 ^f,g^	35.50 ± 0.19 ^e,f^	34.62 ± 0.16 ^d^	33.73 ± 0.21 ^b,c^
	23	36.65 ± 0.21 ^g,h^	36.40 ± 0.17 ^g^	35.74 ± 0.18 ^f^	34.65 ± 0.21 ^d^	33.82 ± 0.20 ^b,c^	35.68 ± 0.18 ^e,f^	34.81 ± 0.22 ^d,e^	33.95 ± 0.17 ^c^	33.41 ± 0.19 ^b^
	30	35.64 ± 0.19 ^e,f^	35.31 ± 0.19 ^e^	35.18 ± 0.15 ^e^	34.71 ± 0.14 ^d^	33.55 ± 0.018 ^b,c^	35.17 ± 0.16 ^e^	34.51 ± 0.14 ^d^	33.56 ± 0.14 ^b^	33.10 ± 0.18 ^a,b^
Gumminess, %	1	2.68 ± 0.02 ^f,g^	2.57 ± 0.02 ^e^	2.53 ± 0.02 ^d,e^	2.48 ± 0.01 ^d^	2.37 ± 0.01^c^	2.46 ± 0.02 ^d^	2.45 ± 0.01 ^d^	2.34 ± 0.01 ^b,c^	2.34 ± 0.02 ^b,c^
	8	2.73 ± 0.01 ^g^	2.68 ± 0.02 ^f,g^	2.60 ± 0.02 ^e,f^	2.54 ± 0.01 ^e^	2.46 ± 0.01 ^d^	2.64 ± 0.02 ^f^	2.62 ± 0.02 ^f^	2.61 ± 0.02 ^e,f^	2.61 ± 0.02 ^e,f^
	15	2.79 ± 0.02 ^g,h^	2.76 ± 0.01 ^g^	2.75 ± 0.01 ^g^	2.75 ± 0.02 ^g^	2.74 ± 0.01 ^g^	2.78 ± 0.02 ^g,h^	2.74 ± 0.01 ^g^	2.71 ± 0.01 ^g^	2.60 ± 0.01 ^e,f^
	23	2.72 ± 0.01 ^g^	2.73 ± 0.01 ^g^	2.88 ± 0.01 ^h,i^	2.89 ± 0.02 ^h,i^	2.83 ± 0.02 ^h^	2.96 ± 0.02 ^i,j^	2.92 ± 0.02 ^i^	2.92 ± 0.01 ^i^	3.03 ± 0.01 ^j^
	30	1.98 ± 0.01 ^a^	2.49 ± 0.01 ^d^	2.98 ± 0.01 ^j^	3.03 ± 0.01 ^j^	2.94 ± 0.01^i^	3.08 ± 0.02 ^k^	3.06 ± 0.01 ^k^	3.02 ± 0.01 ^j^	3.19 ± 0.01 ^l^

The results are presented as the mean of three measurements ± SD (standard deviation). Different letters (^a–o^) designate statistically different results (*p* ≤ 0.05).

**Table 6:** Total polyphenolic compound and antioxidant capacity (DPPH) in vitro evolution of concentrated yogurt with microencapsulated plant extracts during storage.

Parameters	Storage Period, Day	Samples								
		CY	0.15% CYMSE	0.30% CYMSE	0.45% CYMSE	0.60% CYMSE	0.15% CYMRE	0.30% CYMRE	0.45% CYMRE	0.60% CYMRE
TPC in vitro, mg GAE/100g	1	ND	5.24 ± 0.07 ^a,b^	9.47 ± 0.18 ^c^	15.76 ± 0.25 ^f,g^	20.25 ± 0.29 ^i^	4.95 ± 0.03 ^a^	9.91 ± 0.21 ^c^	14.86 ± 0.32 ^f^	19.82 ± 0.40 ^i^
	8	ND	5.32 ± 0.09 ^a,b^	10.14 ± 0.23 ^c^	16.23 ± 0.29 ^g^	20.86 ± 0.35 ^i,j^	5.31 ± 0.08 ^a,b^	10.83 ± 0.18 ^d^	15.76 ± 0.29 ^f,g^	21.86 ± 0.45 ^j^
	15	ND	5.71 ± 0.12 ^b^	10.23 ± 0.18 ^c^	17.08 ± 0.31 ^g^	21.87 ± 0.29 ^j^	5.53 ± 0.12 ^a,b^	11.06 ± 0.25 ^d^	16.45 ± 0.36 ^g^	22.73 ± 0.38 ^j,k^
	23	ND	6.17 ± 0.17 ^b^	11.21 ± 0.26 ^d^	18.72 ± 0.37 ^h^	24.08 ± 0.41 ^k^	6.14 ± 0.19 ^b^	12.09 ± 0.31 ^d,e^	17.87 ± 0.39 ^g,h^	23.59 ± 0.42 ^k^
	30	ND	6.29 ± 0.21 ^b^	11.65 ± 0.19 ^d^	19.23 ± 0.17 ^b^	25.52 ± 0.46 ^l^	6.27 ± 0.17 ^b^	12.44 ± 0.27 ^d,e^	18.21 ± 0.31 ^h^	24.58 ± 0,47 ^k,l^
DPPH in vitro, mM TE/100g	1	0.122 ± 0.004 ^b^	0.185 ± 0.004 ^c^	0.273 ± 0.007 ^e^	0.329 ± 0.017 ^g^	0.347 ± 0.015 ^g,h^	0.186 ± 0.002 ^c^	0.312 ± 0.009 ^f^	0.377 ± 0.012 ^h,i^	0.380 ± 0.015 ^h,i^
	8	0.112 ± 0.002 ^a^	0.192 ± 0.007 ^c^	0.287 ± 0.009 ^e,f^	0.338 ± 0.016 ^g,h^	0.364 ± 0.019 ^h,i^	0.192 ± 0.003 ^c^	0.319 ± 0.011 ^f,g^	0.402 ± 0.014 ^i,j^	0.412 ± 0.015 ^i,j^
	15	0.107 ± 0.003 ^a^	0.195 ± 0.004 ^c^	0.292 ± 0.011 ^f^	0.342 ± 0.012 ^g,h^	0.374 ± 0.016 ^h,i^	0.195 ± 0.002 ^c^	0.336 ± 0.009 ^g^	0.411 ± 0.011 ^i,j^	0.415 ± 0.019 ^i,j^
	23	0.104 ± 0.002 ^a^	0.210 ± 0.004 ^d^	0.303 ± 0.015 ^f^	0.365 ± 0.016 ^h^	0.378 ± 0.021 ^h,i^	0.206 ± 0.004 ^c,d^	0.347 ± 0.012 ^g,h^	0.414 ± 0.015 ^i,j^	0.429 ± 0.012 ^j^
	30	0.101 ± 0.003 ^a^	0.213 ± 0.007 ^d^	0.306 ± 0.012 ^f^	0.382 ± 0.020 ^h,i^	0.392 ± 0.017 ^i^	0.209 ± 0.007 ^c,d^	0.353 ± 0.010 ^g,h^	0.418 ± 0.013 ^i,j^	0.432 ± 0.009 ^j^

The results are presented as the mean of three measurements ± SD (standard deviation). Different letters (^a–l^) designate statistically different results (*p* ≤ 0.05).

## Data Availability

No new data were created or analyzed in this study. Data sharing is not applicable to this article.

## Supplementary Materials

The following supporting information can be downloaded at: https://www.mdpi.com/article/10.3390/antiox12040893/s1, Table S1. Sensory properties (score) evolution of concentrated yogurt with microencapsulated plant extracts during storage.
