# Supplementary material for: The Effect of Aromatic Plant Extracts Encapsulated in Alginate on the Bioactivity, Textural Characteristics and Shelf Life of Yogurt
Source: Antioxidants (Basel). 2023 Apr 6;12(4):893. doi: 10.3390/antiox12040893 (PMC10135706; doi:10.3390/antiox12040893)
Supplement: Supplementary file 1 [file antioxidants-12-00893-s001.zip › antioxidants-2286965-supplementary.pdf]

Supplementary Materials

Table S1. Sensory properties (score) evolution of concentrated yogurt with microencapsulated plant extracts during storage.

Sensory Properties	Storage Period, day	Samples								
		**CY**	**0.15%CYMSE**	**0.30%CYMSE**	**0.45%CYMSE**	**0.60%CYMSE**	**0.15%CYMRE**	**0.30%CYMRE**	**0.45%CYMRE**	**0.60%CYMRE**
Appearance and consistency	1	5.00±0.0 ^{e}	5.00±0.0 ^{e}	5.00±0.0 ^{e}	5.00±0.0 ^{e}	5.00±0.0 ^{e}	5.00±0.0 ^{e}	5.00±0.0 ^{e}	5.00±0.0 ^{e}	5.00±0.0 ^{e}
	8	5.00±0.0 ^{e}	5.00±0.0 ^{e}	5.00±0.0 ^{e}	5.00±0.0 ^{e}	5.00±0.0 ^{e}	5.00±0.0 ^{e}	5.00±0.0 ^{e}	5.00±0.0 ^{e}	5.00±0.0 ^{e}
	15	5.00±0.0 ^{e}	5.00±0.0 ^{e}	5.00±0.0 ^{e}	5.00±0.0 ^{e}	5.00±0.0 ^{e}	5.00±0.0 ^{e}	5.00±0.0 ^{e}	5.00±0.0 ^{e}	5.00±0.0 ^{e}
	23	4.20±0.06 ^{c}	4.24±0.05 ^{c}	5.00±0.0 ^{e}	5.00±0.0 ^{e}	5.00±0.0 ^{e}	4.66±0.04 ^{d}	5.00±0.0 ^{e}	5.00±0.0 ^{e}	5.00±0.0 ^{e}
	30	3.64±0.05 ^{a,b}	3.70±0.03 ^{b}	5.00±0.0 ^{e}	5.00±0.0 ^{e}	5.00±0.0 ^{e}	4.64±0.02 ^{d}	5.00±0.0 ^{e}	5.00±0.0 ^{e}	5.00±0.0 ^{e}
Color	1	5.00±0.0 ^{a}	5.00±0.0 ^{a}	5.00±0.0 ^{a}	5.00±0.0 ^{a}	5.00±0.0 ^{a}	5.00±0.0 ^{a}	5.00±0.0 ^{a}	5.00±0.0 ^{a}	5.00±0.0 ^{a}
	8	5.00±0.0 ^{a}	5.00±0.0 ^{a}	5.00±0.0 ^{a}	5.00±0.0 ^{a}	5.00±0.0 ^{a}	5.00±0.0 ^{a}	5.00±0.0 ^{a}	5.00±0.0 ^{a}	5.00±0.0 ^{a}
	15	5.00±0.0 ^{a}	5.00±0.0 ^{a}	5.00±0.0 ^{a}	5.00±0.0 ^{a}	5.00±0.0 ^{a}	5.00±0.0 ^{a}	5.00±0.0 ^{a}	5.00±0.0 ^{a}	5.00±0.0 ^{a}
	23	5.00±0.0 ^{a}	5.00±0.0 ^{a}	5.00±0.0 ^{a}	5.00±0.0 ^{a}	5.00±0.0 ^{a}	5.00±0.0 ^{a}	5.00±0.0 ^{a}	5.00±0.0 ^{a}	5.00±0.0 ^{a}
	30	5.00±0.0 ^{a}	5.00±0.0 ^{a}	5.00±0.0 ^{a}	5.00±0.0 ^{a}	5.00±0.0 ^{a}	5.00±0.0 ^{a}	5.00±0.0 ^{a}	5.00±0.0 ^{a}	5.00±0.0 ^{a}
Odor	1	5.00±0.0 ^{d}	5.00±0.0 ^{d}	5.00±0.0 ^{d}	5.00±0.0 ^{d}	4.30±0.0 ^{b}	5.00±0.0 ^{d}	5.00±0.0 ^{d}	5.00±0.0 ^{d}	5.00±0.0 ^{d}
	8	5.00±0.0 ^{d}	5.00±0.0 ^{d}	5.00±0.0 ^{d}	5.00±0.0 ^{d}	4.20± 0.02 ^{a,b}	5.00±0.0 ^{d}	5.00±0.0 ^{d}	5.00±0.0 ^{d}	5.00±0.0 ^{d}
	15	5.00±0.0 ^{d}	5.00±0.0 ^{d}	5.00±0.0 ^{d}	5.00±0.0 ^{d}	4.20±0.03 ^{a,b}	5.00±0.0 ^{d}	5.00±0.0 ^{d}	5.00±0.0 ^{d}	5.00±0.0 ^{d}
	23	5.00±0.0 ^{d}	5.00±0.0 ^{d}	5.00±0.0 ^{d}	5.00±0.0 ^{d}	4.18±0.02 ^{a,b}	5.00±0.0 ^{d}	5.00±0.0 ^{d}	5.00±0.0 ^{d}	5.00±0.0 ^{d}
	30	4.48±0.02 ^{c}	5.00±0.0 ^{d}	5.00±0.0 ^{d}	5.00±0.0 ^{d}	4.16±0.03 ^{a}	5.00±0.0 ^{d}	5.00±0.0 ^{d}	5.00±0.0 ^{d}	5.00±0.0 ^{d}
Taste	1	5.00±0.0 ^{g}	5.00±0.0 ^{g}	5.00±0.0 ^{g}	5.00±0.0 ^{g}	3.40±0.04 ^{b}	5.00±0.0 ^{g}	5.00±0.0 ^{g}	5.00±0.0 ^{g}	3.46±0.03 ^{b}
	8	5.00±0.0 ^{g}	5.00±0.0 ^{g}	5.00±0.0 ^{g}	5.00±0.0 ^{g}	3.38± 0.05 ^{a,b}	5.00±0.0 ^{g}	5.00±0.0 ^{g}	5.00±0.0 ^{g}	3.42±0.05 ^{b}
	15	5.00±0.0 ^{g}	5.00±0.0 ^{g}	5.00±0.0 ^{g}	5.00±0.0 ^{g}	3.35±0.06 ^{b,c}	5.00±0.0 ^{g}	5.00±0.0 ^{g}	5.00±0.0 ^{g}	3.40±0.04 ^{b}
	23	4.80±0.05 ^{f}	4.80±0.05 ^{f,g}	5.00±0.0 ^{g}	5.00±0.0 ^{g}	3.34±0.04 ^{a,b}	4.80±0.6 ^{f}	5.00±0.0 ^{g}	5.00±0.0 ^{g}	3.38±0.07 ^{a,b}
	30	3.52±0.06 ^{b,c}	3.68±0.07 ^{c,d}	5.00±0.0 ^{g}	5.00±0.0 ^{g}	3.34±0.06 ^{a,b}	4.58±0.0 ^{e}	5.00±0.0 ^{g}	5.00±0.0 ^{g}	3.35±0.06 ^{a,b}
Overall acceptance	1	5.00±0.0 ^{g}	5.00±0.0 ^{g}	5.00±0.0 ^{g}	5.00±0.0 ^{g}	4.43±0.06 ^{b,c}	5.00±0.0 ^{g}	5.00±0.0 ^{g}	5.00±0.0 ^{g}	4.62±0.01 ^{c}
	8	5.00±0.0 ^{g}	5.00±0.0 ^{g}	5.00±0.0 ^{g}	5.00±0.0 ^{g}	4.40±0.06 ^{b,c}	5.00±0.0 ^{g}	5.00±0.0 ^{g}	5.00±0.0 ^{g}	4.61±0.01 ^{c}
	15	5.00±0.0 ^{g}	5.00±0.0 ^{g}	5.00±0.0 ^{g}	5.00±0.0 ^{g}	4.39±0.01 ^{b}	5.00±0.0 ^{g}	5.00±0.0 ^{g}	5.00±0.0 ^{g}	4.60±0.01 ^{c}
	23	4.75±0.03 ^{d}	4.77±0.01 ^{d}	5.00±0.0 ^{g}	5.00±0.0 ^{g}	4.38±0.01 ^{b}	4.87±0.03 ^{e,f}	5.00±0.0 ^{g}	5.00±0.0 ^{g}	4.60±0.01 ^{c}

30	4.16±0.01 ^{a}	4.35±0.02 ^{b}	5.00±0.0 ^{g}	5.00±0.0 ^{g}	4.38±0.01 ^{b}	4.81±0.01 ^{e}	5.00±0.0 ^{g}	5.00±0.0 ^{g}	4.59±0.02 ^{c}

The results are presented as the mean of three measurements ± SD (standard deviation). Different letters ($^{a-g}$) designate statistically different results ($p \leq 0.05$).
